# Validation of Cervical Cancer Screening Methods in HIV Positive Women from Johannesburg South Africa

**DOI:** 10.1371/journal.pone.0053494

**Published:** 2013-01-11

**Authors:** Cynthia Firnhaber, Nomtha Mayisela, Lu Mao, Sophie Williams, Avril Swarts, Mark Faesen, Simon Levin, Pam Michelow, Tanvier Omar, Michael G. Hudgens, Anna-Lise Williamson, Bruce Allan, David A. Lewis, Jennifer S. Smith

**Affiliations:** 1 Faculty of Health Science Centre, Department of Medicine, University of Witwatersrand, Johannesburg, South Africa; 2 Right to Care, Johannesburg, South Africa; 3 Department of Biostatistics, University of North Carolina, Gillings School of Global Public Health, Chapel Hill, North Carolina, United States of America; 4 Department of OB/GYN, Coronation Hospital, University of Witwatersrand, Johannesburg, South Africa; 5 Cytology Unit Departments of Anatomical Pathology, Faculty of Health Science, University of Witwatersrand and National Health Laboratory Service and Department of Pathology, Johannesburg, South Africa; 6 Institute of Infectious Disease and Division of Medical Virology Department of Clinical Laboratory Sciences University of Cape Town, Cape Town, South Africa; 7 National Health Laboratory Service, Groote Schuur Hospital, Cape Town, South Africa; 8 Centre for HIV and Sexually Transmitted Infections, National Institute for Communicable Diseases National Health Laboratory Services, Johannesburg, South Africa; 9 Department of Epidemiology, Gillings School of Global Public Health, University of North Carolina Chapel Hill, North Carolina, United States of America; 10 Lineberger Comprehensive Cancer Centre, University of North Carolina, Chapel Hill, North Carolina, United States of America; Kinghorn Cancer Centre, Garvan Institute of Medical Research, Australia

## Abstract

**Background:**

HIV-infected women are at increased risk for developing cervical cancer. Women living in resource-limited countries are especially at risk due to poor access to cervical cancer screening and treatment. We evaluated three cervical cancer screening methods to detect cervical intraepithelial neoplasia grade 2 and above (CIN 2+) in HIV-infected women in South Africa; Pap smear, visual inspection with 5% acetic acid (VIA) and human papillomavirus detection (HPV).

**Methods:**

HIV-infected women aged 18–65 were recruited in Johannesburg. A cross-sectional study evaluating three screening methods for the detection of the histologically-defined gold standard CIN-2 + was performed. Women were screened for cervical abnormalities with the Digene HC2 assay (HPV), Pap smear and VIA. VIA was performed by clinic nurses, digital photographs taken and then later reviewed by specialist physicians. The sensitivity, specificity and predictive valves for CIN-2 + were calculated using maximum likelihood estimators.

**Results:**

1,202 HIV-infected women participated, with a median age of 38 years and CD4 counts of 394 cells/mm^3^. One third of women had a high grade lesion on cytology. VIA and HPV were positive in 45% and 61% of women respectively. Estimated sensitivity/specificity for HPV, Pap smear and VIA for CIN 2+ was 92%/51.4%, 75.8%/83.4% and 65.4/68.5% (nurse reading), respectively. Sensitivities were similar, and specificities appeared significantly lower for the HPV test, cytology and VIA among women with CD4 counts ≤200 cells/mm^3^ as compared to CD4 counts >350 cells/mm^3^.

**Conclusions:**

Although HPV was the most sensitive screening method for detecting CIN 2+, it was less specific than conventional cytology and VIA with digital imaging review. Screening programs may need to be individualized in context of the resources and capacity in each area.

## Introduction

Invasive cervical cancer is the third most common cancer among women worldwide [1], with significantly higher incidence rates among HIV-infected than HIV-negative women [2].

Among HIV-infected women, there are no standard guidelines on the optimal methods to screen and treat for cervical cancer in resource-limited countries (RLC). Access to screening in RLC is limited due to financial and personal capacities. The implementation of a cervical cancer screening program with technically appropriate detection methods could reduce morbidity and mortality among HIV-infected women. While alternative screening methods have been evaluated for the detection of high-grade cervical intra-epithelial neoplasia (CIN 2/3) in Africa [3]–[5]; a systematic comparison of the three most common cervical cancer screening methods (Pap smear; visual Inspection of the cervix with 3–5% acetic acid (VIA); and HPV DNA testing) has not been conducted in HIV-infected women in Africa. In addition, none of these studies of HIV-infected women evaluated the performance of the three screening methods stratified by levels of CD4 counts in order to determine if accuracy of these tests vary by immune status.

To determine optimal cervical cancer screening approaches for HIV-infected women, we aimed to compare the sensitivity and specificity of conventional Pap smear screening to that of HPV DNA and VIA testing for the detection of histologically confirmed high-grade cervical neoplasia grade 2 and above (CIN 2+) in 1,202 HIV infected women from Johannesburg, South Africa. We present results of the largest screening study in HIV positive women to date to determine the clinical performance of these three screening methods.

## Methods

### Ethics and Other Approvals

The protocol was reviewed and approved by the Human Ethics Committee (Medical) of the University of the Witwatersrand and, for secondary data analyses, by the University of North Carolina.

### Study Population and enrolment

A total of 1,202 HIV-infected women (18–65 years of age) were recruited from an HIV treatment clinic located in a tertiary government hospital in Johannesburg South Africa. Women were ineligible to participate if they (i) were pregnant, (ii) had previously undergone a hysterectomy or treatment for cervical neoplasia or cancer, iii) were severely ill, or (iv) had signs and/or symptoms suggestive of a sexually transmitted disease (STD). Women were study-eligible following the treatment of any symptomatic STD. Women who were menstruating at study enrollment were asked to return within one to two weeks to participate.

After an educational session was presented on cervical cancer screening in English or Zulu/Sesotho, health workers screened potential eligible women for exclusion criteria, explained study aims, and obtained written informed consent. A medical history was obtained through participant interviews to obtain information on socio-demographic characteristics, antiretroviral therapy status, and other lifestyle factors, including smoking and snuff (traditional chewing tobacco) use, reproductive/menstrual characteristics, sexual history and history of contraceptive use.

### Study related procedures

Each woman was screened for this cross-sectional study using three different methods: i) HPV DNA test (QIAGEN Hybrid Capture 2: HC2), ii) conventional Pap smear cytology, and iii) VIA. During a pelvic examination, the HPV sampling was first conducted using a Digene Cervical Sampler Hybrid Capture-2 (HC-2) brush and placed in standard transport media (STM) (QIAGEN Corporation). HPV DNA laboratory testing was conducted at the University of Cape Town using the Digene Hybird Capture-2 (HC-2) method (QIAGEN), and the HPV laboratory team was blinded to other study results. HPV DNA test results were not used for clinical management.

For a conventional Pap smear diagnosis, cervical exfoliated cells were then collected using a Papette Cervical Cell Collector (Wallach Surgical Devices) and smeared onto cytology slides which were read and analyzed according to Bethesda 2001 guidelines [6]. Women were referred to immediate colposcopy if they had any abnormal cytology diagnosis, including high grade squamous intra-epithelial lesions (HSIL), atypical squamous cells cannot exclude high grade lesion (ASC-H), low grade squamous intra-epithelial lesions (LSIL) and atypical squamous cells of underdetermined significance (ASCUS). To adjust for verification bias [7], twenty-five percent of all women with negative Pap smears and negative VIA were randomly referred for colposcopic biopsy at 12 and 6 locations on the cervix. All cytology smears were analyzed at the National Health Laboratory Services cytology unit.

After Pap smear sampling, VIA was preformed, by applying 5% acetic acid to the cervix followed by a three minute waiting period. Nurses were previously trained at a two week course in Lusaka, Zambia [8]. Visualization of the cervix was conducted and an electronic photographic record was taken using a digital camera. These digital images were used for quality assurance for review by the study specialist physicians. VIA was initially interpreted by the study nurse, and classified as per International Agency for Research on Cancer guidelines (IARC/World Health Organization (WHO)). A VIA was considered positive with the presence of acetowhite lesions, if there were distinct white lesions on the cervix within or close to the transformation zone, covering the cervix, or on a cervical growth [9]. All women with a positive VIA result were referred to colposcopy. During colposcopy, a colposcopic directed biopsy was taken for histological confirmation by an anatomical pathologist. The study cytopathologist and anatomical pathologist were blinded to the VIA, HPV and other study results.

### Quality Assurance

The cytology unit and the Anatomical Pathology Department are accredited by the South African National Accreditation System (SANAS) and undergo regular proficiency testing by the Royal College of Pathologist of Australasia Quality Assurance Programme (RCPA). Internally there is 100% second on minute review by another cytotechnologist of all negative Pap smears and senior cytotechnologist/pathologist review all positive (ASCUS+) cases. Also a there is a cytological/histology review process. Study cytology readings have previously undergone quality assurance by University of North Carolina with 80–85% concordance of results [10], [11].

Discrepant results between cytology and histology resulted in a review of the Pap smear slide. If discrepancy was confirmed, then a repeat colposcopic biopsy was conducted if clinically indicated. For quality assurance (QA) of the VIA technique, the study gynecologist with a medical officer trained in colposcopy reviewed each digital picture and the initial VIA diagnosis of the nurse within two weeks of the VIA procedure. Medical staff was blinded to both the cytology and HPV results at the time of VIA interpretation. If the quality assurance team could not agree on interpretation of VIA results, the digital photos were sent to Professor Parham (blinded to initial readings) for a final diagnosis. HPV testing QA was done per recommendation of the manufacture's guidelines. The final VIA reading used in the analysis was the reading done after review by the doctors at the QA meeting. However calculations using the nurse interpretation for CIN 2+ are also presented.

### Statistical Methods

As all women were not referred for cytology verification by colposcopy, using only the histology results to estimate sensitivity and specificity could lead to biased inference. To correct for this verification bias, we employed the maximum likelihood method proposed by Zhou et al. [12]. Estimates using this method are valid provided that the histology data are missing at random. This assumption means that for women with the same test results, those who were referred to colposcopy were similar to those that were not. Corrected sensitivities and specificities of Pap, HPV DNA, and VIA were estimated using maximum likelihood estimators (MLEs). The positive predictive values (PPVs) and the negative predictive values (NPVs) were also calculated accordingly. 95% confidence intervals (CI) of sensitivities, specificities, PPVs and NPVs were derived based on the asymptotic normality of the MLEs and their asymptotic variances that were estimated by the inverse Fisher information.

For estimation of sensitivity and specificity for CIN-2+ or CIN-3+, Pap smear results were considered negative if the test result was negative, LSIL or ASCUS, and positive if the result was HSIL, ASC-H, or SCC. However additional sensitivity and specificity analyses were done evaluating the Pap smear results comparing negative to positive if the results were ASCUS, LSIL, HSIL, ASC-H or SCC. Estimated sensitivities and specificities of the screening tests, stratified by levels of CD4 counts (≤200 cells/mm^3^, 201–350 cells/mm^3^, 351–500 cells/mm^3^ and >500 cells/mm^3^), HIV viral load ≤400 copies/ml, 401–1000 copies/ml and >1000 copies/ml) and combination antiretroviral therapy (cART) (yes and no), were compared across categories using the standard Z test, assuming independent samples. Agreement between the VIA results by gynecologists and by nurses was measured by a kappa statistic No adjustment was made for multiple comparisons. All statistical analyses were conducted using SAS 9.2 (Cary, North Carolina, USA). The Analysis of VIA for determining CIN 2+ was evaluated by looking at the gynecologists and the nurses VIA interpretation separately.

## Results

### Participant Demographics and Overall Screening Results

A total of 1,202 women were screened between November 2009 and August 2011. Of these, 9 were excluded (6 had inadequate or no cytology; 3 had invalid HPV or VIA results). A total of 1,193 women (98.1% black African) were evaluated, with a median age of 38 years (IQR 32–43) and a median CD4 count of 394 cells/mm^3^ (Interquartile range [IQR] 252.5–572). Approximately 75% (N = 872) of our population had abnormal Pap smears, of which a third (N = 399) of the overall the Pap smears results were high grade lesion by cytology. VIA and HPV were positive in 45% (N = 528) and 61% (N = 727) of participating women, respectively. There were two diagnosed cases of invasive cervical carcinoma. Of the 93.1% (N = 1111) women on cART, 82.9% had undetectable HIV viral loads (≤400 copies/ml).

**Figure 1:**
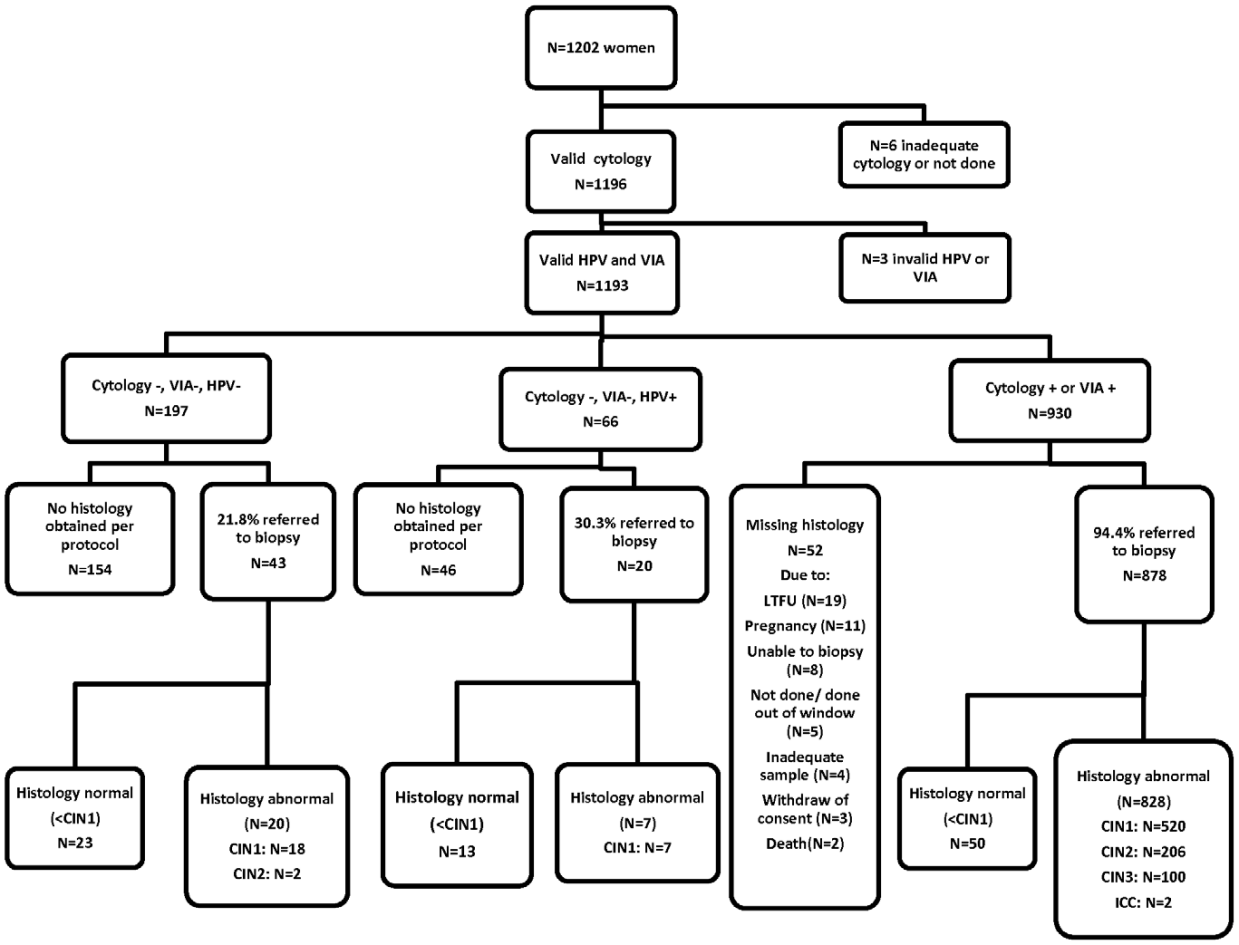
Study Flow Chart.

Figure 1 shows the study flow-chart. Confirmative colposcopy and biopsy was obtained on 94.4% of the study participants with abnormal Pap smear or VIA test results (878/930). The most common reasons for not obtaining a colposcopic biopsy [5.6% (N = 52)] were lost to follow up (N = 19) and pregnancy (N = 11). Approximately 25% (N = 63) of all negative VIA and Pap smear cases also received verification colposcopic biopsies to adjust for verification bias. Table 1 shows the HPV/VIA results stratified by cytology and biopsy results.

**Table 1:** HPV/VIA results stratified by cytology and colposcopy results among 1193 women with a valid HPV test, VIA test and cytology.

*Cytology*	*Colpo not done*	*Negative*	*CIN1*	*CIN2*	*CIN3*	*ICC*	*Total*
	*N (VIA+, HPV+)*	*N (VIA+, HPV+)*	*N (VIA+, HPV+)*	*N (VIA+, HPV+)*	*N (VIA+, HPV+)*	*N (VIA+, HPV+)*	*N (VIA+%, HPV+ %)*
**Negative** (N = 321)	207 (7 ,50)	53 (17 ,19)	55 (30 ,22)	6 (4 ,3)	0 (0 ,0)	0 (0 ,0)	321 (18.1%, 29.3%)
**ASCUS** (N = 31)	2 (0 ,1)	3 (0 ,1)	24 (5 ,5)	2 (0 ,2)	0 (0 ,0)	0 (0 ,0)	31 (16.1%, 29.0%)
**LSIL** (N = 442)	21 (8 ,13)	24 (5 ,9)	338 (127 ,187)	54 (36 ,44)	5 (3 ,5)	0 (0 ,0)	442 (40.5%, 58.4%)
**ASC-H** (N = 30)	3 (2 ,1)	2 (0 ,0)	18 (3 ,9)	4 (2 ,4)	3 (1 ,3)	0 (0 ,0)	30 (26.7%, 56.7%)
**HSIL** (N = 367)	19 (15 ,18)	4 (1 ,3)	109 (67 ,98)	142 (121 ,137)	91 (71 ,89)	2 (2 ,2)	367 (75.5%, 94.6%)
**SCC** (N = 2)	0 (0 ,0)	0 (0 ,0)	1 (0 ,1)	0 (0 ,0)	1 (1 ,1)	0 (0 ,0)	2 (50.0%, 100.0%)
**Total** (N = 1193)	252 (32 ,83)	86 (23 ,32)	545 (232 ,322)	208(163,190)	100 (76,98)	2 (2 ,2)	1193 (44.3%, 60.9%)

Cytology = Cytology Pap smear, HPV = HPV DNA, VIA = Visual inspection with 5% acetic acid, LSIL = Low grade squamous intra-epithelial lesions, ASCUS = Atypical squamous cells of underdetermined significance, HSIL = High grade squamous intra-epithelial lesions, ASC-H = Atypical squamous cells cannot excluded high grade lesion, SCC = Squamous cell carcinoma, CIN1 = Cervical intraepithelial neoplasia grade 1, CIN2 = Cervical intraepithelial neoplasia grade 2, CIN3 = Cervical intraepithelial neoplasia grade 3, ICC = Invasive cervical cancer.

As stand-alone tests, overall sensitivity for the detection of CIN-2+ was the highest for HPV testing (92%), followed by Pap smears (76%) and VIA at 65.4% (nurse interpretation)). However VIA sensitivity was increase to 76% with physician QA review. Specificity for CIN-2+ was the highest for Pap smears (83%), followed by VIA (68% for both doctor and nurse interpretation), and lowest for HPV testing (51%). When CIN3+ was used as the end point, sensitivities were higher, while specificities somewhat lower (∼10%) than observed for CIN-2. Pap smears had a notably higher sensitivity (95%) for CIN-3+ with a corresponding decrease in specificity (73%). Sensitivity for the detection of CIN-3+ increased by 0.7% for VIA, 6.0% for HPV, and 18.7% for cytology as compared to CIN-2+ (Table 2).

**Table 2:** Screening test performance: estimated sensitivity and specificity.

	CIN2+ (N = 310)§		CIN3+ (N = 102)§	
	Sensitivity 95% CI	Specificity 95% CI	Sensitivity 95% CI	Specificity 95% CI
Cytology*	75.8% (70.8–80.8)	83.4% (80.9–85.9)	94.5% (89.8–99.2)	72.7% (70.0–75.3)
VIA (doctor interpretation)	75.5% (70.5–80.4)	68.1% (65.0–71.3)	76.2% (67.9–84.5)	58.9% (56.0–61.9)
HPV	91.9% (88.5–95.3)	51.4% (48.0–54.8)	97.9% (95.0–100)	42.8% (39.8–45.7)
Cytology**	94.8% (90.5–99.2)	35.6% (32.2–38.9)	99.9% (98.8–100)	29.6% (26.9–32.3)
VIA (nurse interpretation)	65.4% (59.7–71.1)	68.5% (65.3–71.7)	68.2% (59.3–77.2)	61.5% (58.6–64.4)
Combined (Or)** Cytology or VIA	89.3% (85.4–93.3)	60.4% (57.1–63.8)	97.4% (93.9–100)	50.7% (47.7–53.6)
Combined (Or)** Cytology or HPV	94.3% (91.3–97.4)	48.5% (45.1–51.9)	99.9% (99.4–100)	39.9% (37.0–42.9)
Combined (Or)** HPV or VIA	95.6% (92.8–98.4)	42.4% (39.1–45.8)	99.0% (97.0–100)	34.7% (31.8–37.5)
Combined (Or)** Cytology or VIA or HPV	96.4% (93.8–99.0)	40.8% (37.4–44.2)	99.9% (99.4–100)	32.9% (30.1–35.8)
Combined (And)** Cytology & VIA	61.3% (55.8–66.8)	91.1% (89.1–93.0)	72.8% (64.1–81.5)	80.9% (78.6–83.3)
Combined (And)** Cytology & HPV	72.6% (67.4–77.8)	86.2% (83.9–88.6)	91.8% (85.9–97.6)	75.5% (72.9–78.1)
Combined (And)** HPV & VIA	71.0% (65.8–76.2)	77.0% (74.1–79.9)	74.6% (66.0–83.1)	67.0% (64.2–69.8)
Combined (And)** Cytology & VIA & HPV	59.3% (53.8–64.8)	91.6% (89.7–93.5)	71.5% (62.6–80.4)	81.8% (79.5–84.2)

§ 94.4% (878/930) of women with abnormal Pap smear/VIA and 25% (63/272) women with negative VIA/Pap smear received a colposcopic biopsy.

CIN2+ = CIN2/CIN3/ICC, CIN3+ = CIN3/ICC, CI = Confidence interval, Cytology = Cytology Pap smear, HPV = HPV DNA, VIA = Visual inspection with 5% acetic acid.

* Cytology negative: normal/LSIL/ASCUS; positive: HSIL, ASC-H, SCC.

** Cytology negative: normal; positive: otherwise.

A combined test in “Combined (Or)” is positive if either/any of the tests is positive. A Combined test in “Combined (And)” is positive if both/all of the tests are positive.

When the results of two screening methods were combined as either test positive, the sensitivity increased to above 89% for detecting CIN 2+ and greater than 97% for CIN 3+. However, there was a corresponding decrease in specificity (Table 2). For CIN2+, the HPV/VIA combination achieved the highest sensitivity (95.6%), but also the lowest specificity (42.4%); the Pap smear/VIA combination had the highest specificity (60.4%). For CIN3+, all combined tests exhibited high sensitivity (>97%); the highest specificity was achieved by the Pap smear/VIA combination (50.7%). When both tests were required to be positive (i.e. HPV and VIA both positive) specificity increased and sensitivity declined compared to a single test (Table 2). Table 3 describes the sensitivity and specificity for the tests when sequentially evaluating the results after one positive test and then adding a second test. Interestingly the most effective testing sequential strategy for detecting CIN 2+ is performing HPV testing after a positive Pap smear. The most specific test was performing a Pap smear after a VIA. Positive and negative predictive value for the three screening methods for both CIN 2+ and CIN 3+ are described in Table 4.

**Table 3:** Sequential screening tests.

	CIN2+		CIN3+	
	Sensitivity 95% CI	Specificity 95% CI	Sensitivity 95% CI	Specificity 95% CI
Cytology+ HPV	96.6% (94.4–98.9)	17.3% (11.0–23.6)	97.8% (94.9–100)	10.4% (6.90–13.9)
Cytology+ VIA	81.7% (77.0–86.5)	46.7% (38.4–55.1)	77.5% (69.3–85.8)	30.4% (25.1–35.6)
HPV+ Cytology	79.8% (75.1–84.5)	71.8% (67.4–76.2)	94.3% 89.5–99.1)	57.2% (53.3–61.1)
HPV+ VIA	78.1% (73.3–82.9)	53.0% (48.1–57.8)	76.7% (68.4–85.0)	42.4% (38.5–46.3)
VIA+ Cytology	81.9% (77.1–86.7)	72.2% (66.8–77.6)	96.1% (91.7–100)	53.6% (49.0–58.3)
VIA+ HPV	94.8% (92.0–97.6)	28.0% (22.6–33.3)	98.6% (95.9–100)	19.7% (16.0–23.4)

**Table 4:** Screening test performance: estimated corrected positive predictive value and negative predictive values.

	CIN2+ (N = 310)		CIN3+ (N = 102)	
	PPV 95% CI	NPV 95% CI	PPV 95% CI	NPV 95% CI
Cytology*	64.5% (59.7–69.3)	89.7% (87.3–92.1)	25.7% (21.3–30.1)	99.2% (98.6–99.9)
VIA	48.5% (44.0–53.0)	87.5% (84.6–90.3)	15.7% (12.4–18.9)	96.1% (94.6–97.6)
HPV	42.9% (39.0–46.8)	94.1% (91.5–96.7)	14.6% (11.9–17.3)	99.5% (98.8–100)
Combined Cytology/VIA	47.3% (43.4–51.3)	93.4% (90.9–96.0)	16.6% (13.6–19.5)	99.5% (98.8–100)
Combined Cytology/HPV	42.2% (38.5–45.8)	95.5% (93.1–98.0)	14.3% (11.7–16.9)	100% (99.9–100)
Combined HPV/VIA	39.8% (36.3–43.3)	96.0% (93.4–98.6)	13.2% (10.8–15.6)	99.7% (99.1–100)
Combined Cytology/VIA/HPV	40.8% (37.3–44.3)	96.4% (93.7–99.0)	13.8% (11.3–16.2)	100% (99.8–100)

CIN2+ = CIN2/CIN3/ICC, CIN3+ = CIN3/ICC, PPV = Positive predictive value, NPV = Negative predictive value, CI = Confidence interval, Cytology = Cytology Pap smear, HPV = HPV DNA, VIA = Visual inspection with 5% acetic acid.

* Cytology negative: normal/LSIL/ASCUS; positive: HSIL, ASC-H, SCC.

### Screening results stratified by HIV disease status

We compared the operating characteristics of the three screening methods by immune status of the patients using CD4 counts. Standard Z tests comparing the sensitivities and specificities across strata of CD4 counts show that there was no significant difference in sensitivities within different levels of CD4 counts. However, specificity appeared significantly lower for women with CD4 counts ≤200 cells/mm^3^ compared to CD4 counts >350 cells/mm^3^ (*p*<0.001 for HPV, *p*<0.001 for cytology, *p* = 0.002 for VIA) (Table 5). Comparing the test performance characteristics between women with HIV viral load ≤1000 and >1000 copies/ml, or by cART status was not conducted due to the small proportion of women who were not on cART (6.9%) and because the vast majority had suppressed HIV viral loads.

**Table 5:** Screening test performance for CIN2+: estimated corrected sensitivity and specificity by CD4 count (cells/mm3).

	CD4	CIN-2+ cases	Sensitivity	p value*	Specificity	p value*
Cytology**	≤200	58	74.0% (62.3–85.6)		74.3% (65.7–83.0)	
	201–350	103	82.1% (74.5–89.7)	0.086	81.7% (76.6–86.7)	0.033
	>350	146	71.9% (64.3–79.5)	0.778	85.9% (82.8–88.9)	<0.001
HPV	≤200	58	92.7% (85.2–100)		31.6% (22.3–40.9)	
	201–350	103	97.4% (93.7–100)	0.038	41.7% (35.3–48.1)	0.024
	>350	146	88.8% (83.3–94.4)	0.687	59.7% (55.4–64.0)	<0.001
VIA	≤200	58	72.5% (60.8–84.1)		60.6% (50.9–70.3)	
	201–350	103	83.3% (75.9–90.7)	0.032	64.0% (57.7–70.3)	0.25
	>350	146	71.3% (63.7–78.9)	0.683	71.1% (67.2–75.1)	0.002

* This is the p value comparing each group with the reference group (≤200 cells/mm^3^).

** Cytology negative: normal/LSIL/ASCUS; positive: HSIL, ASC-H, SCC.

### Quality Assurance

14% of all participants had a discrepancy between cytology and histology results. Verification biopsies were done on 25% of all women with negative VIA and Pap smears. Only 3% (2/63) of these verification histology results were positive, resulting in two CIN 2 cases. Histological review of the follow-up Loop Electrical Excision Procedure (LEEP) results for these two CIN-2 cases revealed only minimal changes consistent with HPV infection. No neoplasia was found. There was substantial agreement between the VIA readings of the nurse and that of the doctor [kappa statistic = 0.69 (95% CI 0.64–0.73)].

## Discussion

This analysis of just over 1,200 HIV-infected women represents, to our knowledge, the largest comparative screening study of HIV-infected women, comparing the screening performance of three cervical cancer screening methods to detect histological CIN 2+ endpoints. We observed a notably high rate of positive test results, including 33% HSIL, 61% HPV DNA positivity, and 45% VIA positivity. All three screening methods had sensitivities of >65% for determining CIN 2+ disease in HIV positive women, with variations in specificity for CIN-2 ranging from 83% for cytology to 51% for HPV testing. Test sensitivities were similar across strata of CD4 counts, while the specificities of all the screening methods decreased with immune suppression as measured by CD4 counts.

The observed HSIL prevalence in this study (33%) is almost double than that previously reported from a different cohort of 1010 HIV-infected women from the same clinic (18%) [11]. The vast majorities of study participants on this study were on cART and were not significantly immunosuppressed. However, these women had initiated cART when their CD4 counts were below 200cells/mm^3^, as per the South African HIV treatment guidelines and thus had a prior history of significant immunosuppression [13]. Our higher observed prevalence of HSIL may be attributable to women living longer due to cART use. These cytology results expose the burden of high grade cervical dysplasia, and highlight the significant public health problem of cervical dysplasia in HIV-infected women in South Africa.

Cytology based screening (via the Pap smear) is the only screening method that has been shown to reduce mortality in many places in the world including middle and lower resource countries such a Colombia, Chile and Vietnam [14]–[16]. The performance of Pap smear screening for CIN 2+ among women in general population-based studies internationally has ranges for both sensitivity (40–86%) and specificity (88–99%) [17]–[19]. Our study results were within these ranges.

At present, there are only three studies evaluating different screening methods for the detection of histological CIN 2+ in HIV infected women in Africa. None of these studies conducted had direct comparisons of these three screening methods. In Nigeria (N = 205), VIA was found to have a sensitivity and specificity of 76% (95% CI 52–91%) and 83% (95% CI 77.0–88.0%), respectively [3]. In Kenya, VIA was performed in 150 HIV infected women and was found to have a sensitivity 69.6% (CI 55.1–81%) and specificity of 51% (CI 41.5–60.4%) [4]. A total of 956 HIV infected women in Cape Town were studied, with an observed sensitivity of VIA of 64% and HPV of 94% for detecting CIN2+. Cytology was not evaluated in this study [5] The range of average age of HIV infected women in these studies was somewhat similar to ours from 34 years (Kenya, Nigeria) to 40 years in the Cape Town cohort.

A very similar study to ours was performed with 303 HIV positive women in India, and showed similar sensitivity/specificity of VIA for CIN-2+ detection of 80/82% respectively [20]. This Indian study showed a similar relationship between CD4 count and specificity of the screening tests to our study. Women with lower CD4 counts (<350 cells/mm3) had lower specificity for VIA and cytology [20]. Overall, the sensitivity of VIA for CIN-2+ detection in HIV infected women within the five studies (including our present study) has ranged from 64–80% and the specificity from 76–83% [3]–[5], [20].

The results of VIA in HIV negative women in two large meta-analyses showed that the range of sensitivity of VIA was relatively similar at 79%–80%. The range for specificity for VIA was slightly higher (85–92%) in HIV-negative than in HIV infected women [21], [22].

The VIA sensitivity for CIN 2+ is within the range of the Pap smear sensitivity in this study, although the observed specificity is not as high as the Pap smear in HIV positive women. However for reasons that are unclear, the Pap smear had a much higher sensitivity then the VIA (which remained unchanged) for detecting CIN 3+. Sequential HPV testing as seen in Table 3 improves this senstitivity to 98.6%. However, VIA offers the advantage of being relatively inexpensive to implement where access to cytology based systems are not available, offers the possibility of same visit treatment and can be performed by a nurse after a short training period (often two weeks). An additional advantage of VIA is that the nurse can immediate treat an appropriate lesion by cryotherapy. This allows the woman to be screened and treated in one clinic visit decreasing the risk of lost to follow up of these high risk women and reduces the number of clinic visits for overwhelmed and under-capacitated clinics [8].

High risk HPV DNA was present in 61% of participants in our study which is somewhat higher than that observed among 956 HIV-infected women from Cape Town (46%) [5], yet consistent with prevalence rates of oncogenic HPV among other HIV-infected women worldwide [23]. HPV typing in our study was sensitive for high-grade detection (92% for CIN-2+, 98% for CIN-3+), although specificity was lower (51.1% for CIN-2, 42.8% for CIN-3). HPV testing could be used in combination with either VIA or Pap smear to increase specificity and possibly reduce follow-up procedures such as colposcopy. Adding HPV testing after a positive Pap smear or VIA increased the sensitivity of these tests to the levels slightly for CIN 2+ above HPV testing alone but the addition of HPV testing to VIA did improve the reduce sensitivity of VIA for CIN 3+. However adding the HPV testing significantly diminished the specificity of the Pap smear and VIA screening tests alone. HPV testing offers the possibility of self-testing with a relatively high sensitivity for CIN-2+ in HIV negative women [24]. However, HPV testing is, at present, relatively expensive and requires skilled laboratory services. A new HPV test called Care-HPV (QIAGEN/PATH) which is less expensive and simpler to perform will hopefully be available soon for commercial use [25].

South Africa has one of the highest HIV prevalence rates in the world [26]. As HIV infected women have improved access to cART due to government and donor programs, women are living relatively longer lives [27], [28] and thus are at a higher risk of progression from CIN 2/3 to invasive cervical carcinoma. In our study the specificity of HPV, VIA and cytology, appeared to be lower among women with lower CD4 counts, and the reason for this observation is unclear. One speculation might be that in women with lower CD4 counts there maybe have other infections causing interference with the tests decreasing specificity. Understanding if and why immunosuppression would lead to lower test specificity is intriguing and requires further evaluation. These results need to be replicated, and further research is also needed evaluating the effect of HIV disease status on screening. Given that cART has recently been shown to be potentially effective in decreasing the rate of progression to HSIL or more severe [29]–[31], randomized controlled trials will be required to examine the effect of starting ART earlier on the incidence of HSIL lesions.

In terms of study strengths, we implemented intense QA measures to ensure reliable visual inspection results, which included weekly meetings to review all digital photos by a team of study personnel. This helped ongoing education of the nursing staff and likely contributed to the good correlation between the nurses' and doctors' VIA readings. In addition to skills resources needed, the QA of VIA also required significant infrastructure (i.e. electricity, computers and projectors). Such resources may not be available in other areas of South Africa or other RLC. However, this QA model to optimize VIA results was adapted from the program in Lusaka, Zambia. For rural sites in resource limited countries, this essential QA model could potentially be modified by sending pictures via “memory sticks”/CDs or through cell phones to skilled personnel. We have been able to achieve this model in rural South Africa.

Study limitations relate to the study review and intense QA which might be difficult to successfully replicate in other public clinic and non-academic environments. Participant files were reviewed to ensure results and follow-up visits were achieved. In busy under-resourced clinics in RLC, these types of review and meetings may be extremely difficult to thoroughly implement. Further, most of our patients were on effective cART so precluding our ability to determine the effect of cART and HIV viral load on screening.

Cervical cancer screening in HIV positive women is an urgent public health requirement which demands immediate attention and coordinated efforts of both national and local governments. Our study results indicate that all three screening modalities (HPV, VIA, cytology) are viable alternatives for consideration as screening options in different programmatic settings, which is important as a “one size fits all approach” may not to work. The decision of which screening modality to implement will be influenced by cost, patient population, availability of skilled human resource and laboratory capacity. Within this decision process, quality assurance needs to be considered at all stages of the program. Careful consideration and evaluation will be needed to determine for the best screening approach for a country and maybe for different geographical settings within a country.
